# Enteral Nutrition in Neonates on Inotropic Support Admitted to a Tertiary Neonatal Intensive Care Unit: A Prospective Cohort Study

**DOI:** 10.7759/cureus.69705

**Published:** 2024-09-19

**Authors:** Deepika Mittapalli, Anil K Sajjan, Siddu Charki, Mallanagouda Patil

**Affiliations:** 1 Pediatrics, Shri B. M. Patil Medical College Hospital and Research Centre, BLDE (Deemed to be University), Vijayapura, IND

**Keywords:** enteral feeds, feed tolerance, inotropes, neonatal intensive care unit (nicu), neonates

## Abstract

Background

Early initiation of enteral feeding in neonates on inotropic support may improve clinical outcomes compared to intravenous fluids, but the safety and optimal inotrope levels for enteral nutrition remain unclear. This study aims to assess the early initiation of enteral feeding versus intravenous fluids in newborns on inotrope support and to compare the clinical outcome in terms of hospital stay, morbidity, and mortality in neonates with enteral feed versus intravenous (IV) fluid group on inotropes. It also focuses on evaluating the safety of enteral nutrition in neonates with inotropic support and determining the cutoff levels of the inotropes at which enteral feed is well tolerated.

Methodology

A prospective cohort study was conducted at a tertiary care center in northern Karnataka from June 2022 to December 2023. Neonates born after 28 weeks or more of gestation or weighing more than 1000 grams with fluid nonresponsive shock were enrolled for the study. Neonates with gastrointestinal comorbidities or birth weight below 1000 grams, younger than 28 weeks of gestation, and with lethal congenital malformations were excluded. Eligible neonates were enrolled by the investigator into enteral and intravenous fluid groups at the clinical discretion of the treating neonatologist. Enteral feeding commenced with expressed breast milk or milk from milk bank after 6 hours of stable circulation while the intravenous fluid group received no feed initially. Inotropes were tapered upon stable circulation with feed volumes adjusted based on tolerance. The data obtained was entered into a Microsoft Excel sheet and statistical analyses were performed using IBM SPSS Statistics software, version 20. The results were presented as mean, standard deviation counts, percentages and diagrams. For normally distributed continuous variables between the two groups, they were compared using an independent sample t-test. For not normally distributed variables, the Mann-Whitney U test was used. Categorical variables between the two groups were compared using the chi-square test and Fisher's exact test and p<0.05 was considered statistically significant.

Results

The study included 142 neonates (71 per group). The enteral nutrition group had a higher percentage (62; 87.32%) of improved and discharged neonates than the intravenous fluid group (56; 78.87%). The mean time to reach full feeds was significantly lower in the enteral nutrition group (6.04 days) as compared to the intravenous fluid group (9.78 days), with a p-value of < 0.0001. Similarly, the duration of neonatal intensive care unit (NICU) stay was significantly shorter for enteral nutrition group (7.38 days) compared to intravenous fluid group (11.37 days), also with a p-value of < 0.0001. Three patients (4.22%) chose to get discharged against medical advice in the enteral nutrition group while five patients (7.04%) did the same in the intravenous fluid group. The death rate was also higher among intravenous fluid group (10; 10.08%) compared to enteral nutrition group (6; 8.45%). Independent samples t-test showed significant differences in time to reach full feed and duration of NICU stay between cases and controls.

Conclusion

Neonates receiving enteral feeds had significantly shorter NICU stays, suggesting improved clinical management. Adrenaline (0.1 mcg/kg) with dobutamine (10 mcg/kg) emerged as the optimal inotrope combination for feed tolerance.

## Introduction

Malnutrition has long been associated with poor clinical outcomes, particularly in developing countries where its prevalence is alarmingly high. The compounding effects of malnutrition and disease processes in these regions exacerbate the health challenges faced by vulnerable populations, making early nutritional support crucial [1]. Among the various methods of nutritional support, the enteral route is generally preferred for patients with a functional gastrointestinal (GI) tract. Evidence suggests that critically ill patients receiving parenteral nutrition experience longer hospital stays and have a higher risk of sepsis [2].

Complications such as villi flattening, mucosal thinning, and bacterial translocation from the gut lumen into the systemic circulation can activate various inflammatory mediators [3-9]. The high cost of parenteral nutrition further complicates its use, making it an unaffordable option for many. In contrast, enteral nutrition offers several advantages, including better substrate utilization, reduced infection rates, shorter hospital stays, decreased mortality, and the early establishment of gut flora [10]. Breast milk, in particular, has many benefits and facilitates early initiation of enteral nutrition in newborns [11,12].

Despite these advantages, there is a notable lack of systemic research and clinical trials on various aspects of enteral nutrition in neonates, particularly neonates who require inotropic support due to hemodynamic instability. This knowledge gap poses a major challenge in the development of evidence-based practice guidelines. To address this gap, we hypothesized to examine the effect of early initiation of enteral nutrition in neonates who require inotropic support versus neonates only on intravenous (IV) fluids admitted in neonatal intensive care (NICU). Furthermore, this study aims to determine the inotropic threshold at which enteral nutrition is well tolerated and provide important insights for optimizing nutritional support in hemodynamically unstable neonates.

## Materials and methods

A prospective cohort study was conducted at a tertiary care center in northern Karnataka, spanning from June 2022 to December 2023. In this study, 142 neonates were included, out of which 71 were put in the enteral feed group (cases) and 71 in the intravenous fluid group (controls).

Inclusion and exclusion criteria

All neonates born after 28 weeks of gestation or >1000 grams with fluid nonresponsive shock, requiring inotropic support were enrolled. Newborns associated with co-morbid conditions of the gastrointestinal tract (GIT), birth weight <1000 g, gestational age <28 weeks, and lethal congenital malformations were excluded from this study.

Methodology

After collecting a detailed history and proper clinical examination, neonates with signs of reduced perfusion/shock, lethargy, comatose, respiratory distress, tachypnoea or apnea, pallor, cyanosed or convulsions, tachycardia, capillary refill time >3 seconds, poor pulse volume, low blood pressure, and decreased urine output were identified. Eligible neonates were enrolled by the investigator based on the clinical discretion of the treating neonatologist into either the enteral group (cases), which received tube feeding with expressed breast milk or milk from a milk bank after adequate circulation [13] (defined as adequate balance between oxygen delivery and oxygen consumption on both systemic and regional levels), for 6 hours or the intravenous fluid group (controls), which did not receive any feed initially. Feed volumes in the enteral group were gradually increased once tolerance was established, aiming for 140-160 mL/kg/day. Continuous monitoring assessed the hemodynamic status and identified signs of feed intolerance (vomiting/abdominal distension >2 cm/ blood‑stained residues/residual feed >50%) was looked for. Inotropes were tapered over 12-24 hours upon stable circulation. This study's outcomes included improvement and discharge, discharge against medical advice, or mortality.

Statistical analysis

G*Power ver. 3.1.9.4 software was used for sample size calculation. The proportion of discharged neonates in the enteral group was 88.29% and in the IV fluid group was 69.6%. Hence this study required a sample size of 142 (with 71 in each group, assuming equal group sizes) to achieve a power of 80% for detecting differences in proportions between two independent groups using an exact test for inequality with a 5% level of significance. Data was entered into MS Excel spreadsheet (Microsoft, Redmond, WA). IBM SPSS Statistics, version 20 (IBM Corp., Armonk, NY) was used for data analysis. Descriptive statistics were elaborated in the form of means/standard deviations for continuous variables, and frequencies and percentages for categorical variables. Data was presented graphically wherever appropriate for data visualization using histograms, column charts for continuous data, and bar charts/pie charts for categorical data. Group comparisons for categorically distributed data were made using the chi-square test. Comparisons for continuous data were done by independent sample t-test. The receiver operating characteristic curve (ROC) curve was plotted to identify the cutoff value for inotrope dosage determination. For statistical analysis to establish the ROC curve, dobutamine dosage alone was considered for cases. Sensitivity, specificity, and Youden’s index were calculated using the coordinates from the ROC curve. Statistical significance was kept at p < 0.05.

## Results

In this study, 142 neonates were included, out of which 71 were included in the enteral feed group (cases) and 71 in the intravenous fluid group (controls). Demographic analysis revealed preterm births were higher in cases (66.2%) than in controls (60.6%) with no statistical significance (p=0.486). Delivery mode showed a slightly higher cesarean section rate among cases (60.6%) versus controls (57.7%) with p>0.05. Maternal risk factors such as pregnancy-induced hypertension, gestational diabetes mellitus, eclampsia, oligohydramnios, hypothyroidism, premature rupture of membranes, and antepartum hemorrhage were more common in controls (59.2%) than cases (52.1%) with no statistical significance. Clinically, cases had higher Apgar scores at 1 minute (64.7% scored 7) compared to controls (53.9% scored 6), which was not statistically significant. Respiratory distress syndrome (RDS) was the most frequent diagnosis in the cases (40.8%) and asphyxia was more common in controls (29.6%). Non-invasive respiratory support was observed to be higher in the cases (61; 85.9%), whereas controls had higher invasive support (22; 31.0%) (Table 1).

**Table 1:** Demographic data and baseline clinical parameters of both groups p-value<0.05 is considered as statistically significant

Parameter	Enteral Nutrition Group (Cases n=71)	Intravenous Fluid Group (Controls n=71)	p-value
Gender			
Male	44	42	-
Female	27	29	-
Gestational age (weeks)			
Preterm	47	43	0.486
Term	24	28	
Place of delivery			
Inborn	35	35	1.00
Outborn	36	36	
Birth weight (grams)			
<2 kg	20	211	0.441
>3 kg	7	12	
2-3 kg	44	38	
Apgar score			
1 min (no. of newborns in parenthesis)	7 (33), 6 (29)	7 (18), 6 (34)	0.295
Mode of delivery			
C-section	43	41	0.773
Vaginal	28	30	
Maternal risk factors			
Yes	37	42	0.398
None	34	29	
Diagnosis			
Respiratory distress syndrome	29	21	0.462
Asphyxia	13	21	
Meconium aspiration syndrome	15	13	
Sepsis	14	16	
Respiratory support			
Non-invasive support	61	49	0.006
Invasive support	10	22	
Sepsis			
Culture-positive proven sepsis	8	11	0.467
Probable sepsis	63	60	

Among the cases, 65 neonates (91.5%) did not experience neonatal complications such as feed intolerance (vomiting/abdominal distension >2 cm/ blood‑stained residues/residual feed >50%) while six neonates (8.40%) experienced such complications. In contrast, among the controls, 68 neonates (95.77%) did not experience neonatal complications, while three neonates (4.22%) did. The chi-square test resulted in a non-significant finding (p = 0.301), indicating that the occurrence of neonatal complications did not significantly differ between the two groups (Table 2).

**Table 2:** Comparison of neonatal complications among cases and controls

Neonatal Complications	Cases	Controls
No	65 (91.5%)	68 (95.77%)
Yes	6 (8.40%)	3 (4.225%)

Outcomes showed more improvement and discharges in cases (62; 87.3%) than in controls 56(78.87%) and discharge against medical advice (DAMA) was observed to be higher in controls (5; 7.04%) than in cases (3; 4.22%). Similarly, deaths were higher in controls (10; 10.08%) than cases (6; 8.45%). Significant differences were noted in the time to reach full feeds (6.04 days for cases and 9.78 days for controls) with a p-value of <0.0001 and NICU stay duration was 7.38 days for cases and 11.37 days for controls with a p-value of <0.0001 (Table 3).

**Table 3:** Clinical findings and outcomes with p-values DAMA: Discharge against medical advice; NICU: neonatal intensive care unit; SD: standard deviation p-value<0.05 is considered as statistically significant

Parameter	Group 1 (Cases)	Group 2 (Controls)	P-value
Time to reach full feeds in NICU (days)	Mean: 6.04 (SD = 1.04)	Mean: 9.78 (SD =1.15)	<0.0001
Duration of NICU stay (days)	Mean: 7.38 (SD = 2.05)	Mean: 11.37 (SD = 2.82)	<0.0001
DAMA	3	5	0.406
Death	6	10	
Improvement and discharge	62	56	

**Figure 1:**
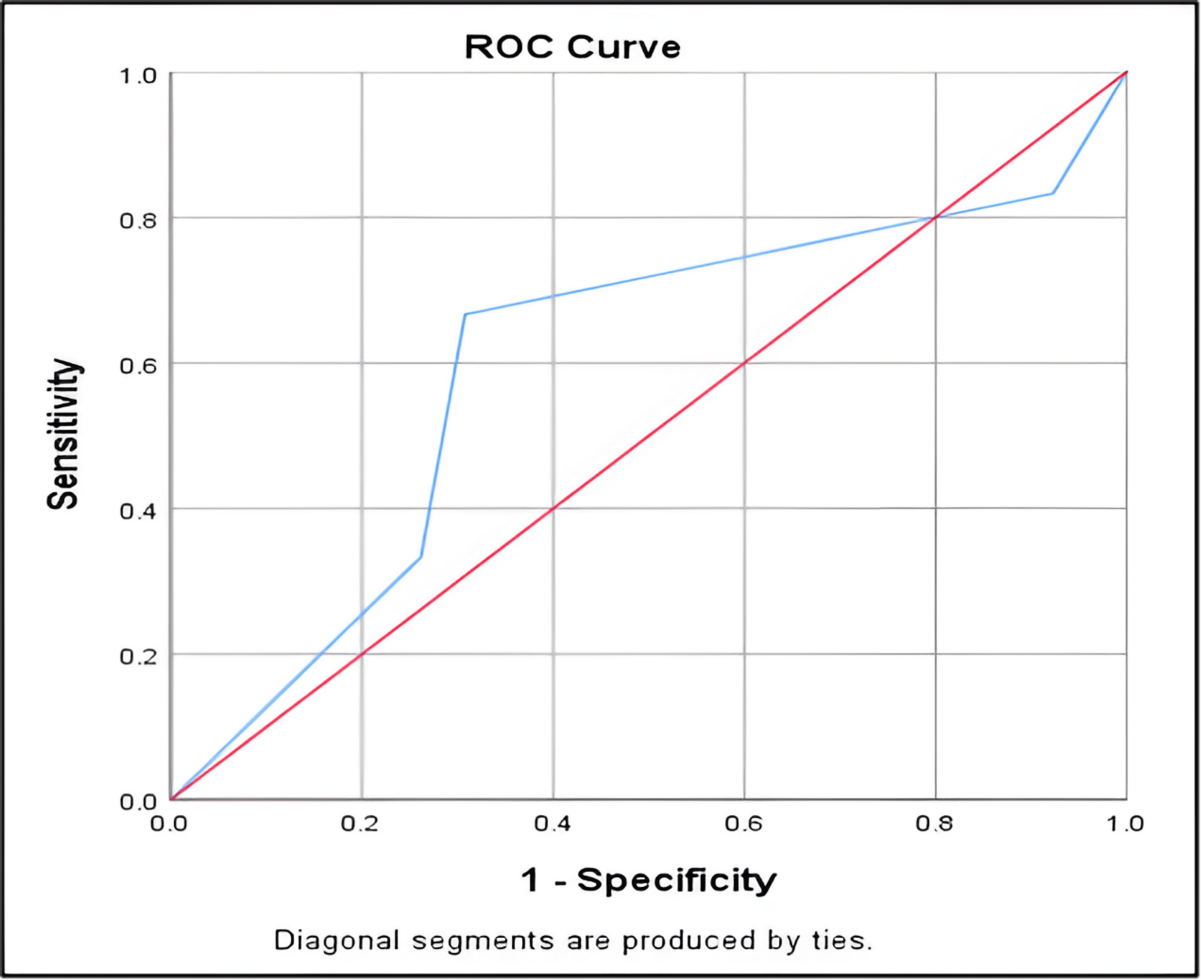
Area under the curve for inotrope dosage for feed tolerance ROC: Receiver operating characteristic curve

The area under the curve for inotrope dosage for feed tolerance among the newborns was found to be 0.6, which indicates that it is a satisfactory indicator of feed tolerance at the inotrope dosage of adrenaline (0.1mcg/kg) + dobutamine (10mcg/kg) (Figure 1).

From the ROC curve, dobutamine (5mcg/kg) + dopamine (5mcg/kg) demonstrates higher sensitivity but lower specificity compared to adrenaline (0.1mcg/kg) + dobutamine (10mcg/kg), which shows a balance with 66.67% sensitivity and 69.23% specificity. Dobutamine (10mcg/kg) + dopamine (10mcg/kg) and adrenaline (0.2mcg/kg) + dobutamine (15mcg/kg) exhibit lower sensitivity and higher specificity. The latter achieves perfect specificity but lacks sensitivity. Therefore, the choice of inotrope regimen should consider these trade-offs in clinical decision-making for optimizing patient outcomes (Table 4).

**Table 4:** A comparison of different inotrope dosages based on their sensitivity, specificity, and Youden’s index as diagnostic performance metrics mcg/kg: Micrograms/kilogram

Inotrope Dosage	Sensitivity %	Specificity%	Youden’s index
Dobutamine (5mcg/kg) + dopamine(5mcg/kg)	83.33	7.69	-8.97
Adrenaline (0.1mcg/kg) + dobutamine (10mcg/kg)	66.67	69.23	35.90
Dobutamine (10mcg/kg) + dopamine(10mcg/kg)	33.33	73.85	7.18
Adrenaline (0.2mcg/kg) + dobutamine (15mcg/kg)	0.00	100.00	0.00

## Discussion

This study had a higher number of male children in both the cases (44; 62.0%) and controls (42; 59.2%), which aligns with the findings of Rao et al. [14]. This gender disparity is common in neonatal studies, potentially due to biological and sociocultural factors. The gestational age distribution also showed a higher incidence of preterm births in cases (47; 66.2%) compared to controls (43; 60.6%) similar to the findings of Rao et al. [14], who reported preterm births to be 65.2% and 60.2%, respectively. Both studies observed a predominant birth weight range of 2-3 kilograms, though the direct comparison was challenging due to differences in reporting specifics. The mode of delivery indicated slightly higher cesarean section (LSCS) rates among cases in our study 43 (60.6%) compared to Rao et al. [14] (60.2%), with controls also showing a similar trend 41 (57.7% vs. 55.5%). This might reflect variations in clinical practices and decision-making processes in managing high-risk pregnancies.

Maternal risk factors such as pregnancy-induced hypertension, gestational diabetes mellitus, eclampsia, oligohydramnios, hypothyroidism, premature rupture of membranes, ante-partum hemorrhage were comparable between the studies. Our controls showed slightly higher occurrences of maternal risk factors (59.2%) compared to cases (52.1%), akin to the trend in Rao et al. [14] (58.5% in controls vs. 53.8% in cases). Clinical characteristics, such as Apgar scores at 1 minute, showed higher scores more frequently in controls in both studies, emphasizing the importance of early neonatal assessment.

In terms of clinical outcomes, both studies highlighted significant differences in the prevalence of conditions like respiratory distress syndrome (RDS) and perinatal asphyxia between cases and controls, with slight variations in percentages. Our study found significant differences in respiratory support modalities with p-value of 0.016 with no statistical significance, with non-invasive support being more common in cases 61 (85.9%) compared to controls 49 (69.0%). Rao et al. [14] reported similar trends. Our study determined that a combination of adrenaline at (0.1 mcg/kg/min) and dobutamine at (10 mcg/kg/min) was optimal for predicting feed tolerance, achieving a balance between sensitivity (67%) and specificity (69%) when assessed using receiver operating characteristic (ROC) curve analysis. Rao et al. [14] identified dopamine at (10 mcg/kg/min) and dobutamine at (7.5 mcg/kg/min) as optimal, with a sensitivity of 61.5% and specificity of 77%, in contrast, the probable reason for better tolerance of feeds in neonates on inotropic support of adrenaline at 0.1 mcg/kg and dobutamine at 10 mcg/kg in our study could be because dobutamine tends to improve gastrointestinal mucosal blood flow when given at (10 mcg/kg/min) whereas adrenaline has a lesser vasoconstrictive effect when given at (0.1mcg/kg/min). The uniqueness of these values apart from other studies points towards the demand for personalized inotrope control guided by unique clinical situations.

The study conducted by Mancl and colleagues [15] sheds light on the lack of data regarding the administration of nutrition (EN) to patients who received intravenous (IV) vasopressor therapy for hemodynamic support. Their findings indicated that providing EN was well tolerated and safe for patients receiving IV vasopressor support at levels of (12.5 mcg/min) of norepinephrine or lower. This suggests that maintaining EN could be beneficial for patients with moderate vasopressor needs. However, more research is necessary to confirm these results in patient populations and across levels of vasopressor support.

Yang and team highlighted the debate surrounding feeding in hemodynamically unstable critically ill patients, citing concerns about ischemic complications and variations in patient responses to feeding approaches and vasoactive agents [16]. Zaloga et al. [17] noted that vasopressors like dobutamine tend to enhance gastrointestinal mucosal blood flow, whereas dopamine and norepinephrine show variable effects depending on the patient's condition. These insights emphasize the need for careful monitoring and individualized approaches to enteral nutrition in these patients.

Clinical implications

Our study's findings contribute to the growing body of evidence supporting the early initiation of enteral nutrition in newborns with hemodynamic instability while on inotropes. While the risk of feeding-associated bowel necrosis in neonates on high-dose vasopressors remains a concern, this study contributes to improving the current practice through advocating the early initiation of enteral nutrition in neonates on inotropic support with a better understanding of the interplay between gut health, systemic hemodynamic stability, and enteral nutrition.

Strengths and limitations

Our study highlights the importance of the administration of enteral nutrition during the provision of low doses of inotropes with close monitoring of feed tolerance with very minimal risk of bowel necrosis. The study's sample size of 71 cases and 71 controls may limit the generalizability of findings to larger populations or different demographic groups. There is a potential for selection bias, as the study population was likely drawn from a specific hospital or region and was based on the clinical discretion of the neonatologist at admission, which may not fully represent the broader population. In future research prospects, it would be better to consider antenatal dopplers along with severity of fetal hypoxia for better tolerance of enteral nutrition in neonates on inotropic support.

## Conclusions

Neonates on inotropic support with early initiation of enteral feeds had a significantly shorter duration of NICU stay and the time taken to reach full feeds was less, suggesting improved clinical outcomes. Adrenaline (0.1 mcg/kg) + dobutamine (10 mcg/kg) emerged as the optimal inotropic combination for better tolerance of enteral nutrition in hemodynamically unstable neonates.
